# Glioblastoma-Derived Exosomes as Nanopharmaceutics for Improved Glioma Treatment

**DOI:** 10.3390/pharmaceutics14051002

**Published:** 2022-05-06

**Authors:** Hyeji Lee, Kanghye Bae, Ah-Rum Baek, Eun-Bin Kwon, Yeoun-Hee Kim, Sung-Wook Nam, Gang Ho Lee, Yongmin Chang

**Affiliations:** 1 Department of Medical Science, School of Medicine, Kyungpook National University, Daegu 41944, Korea; pba04052@naver.com (H.L.); hjbkh980708@naver.com (K.B.); 2 Institute of Biomedical Engineering Research, Kyungpook National University, Daegu 41405, Korea; baxun@naver.com; 3 Korean Medicine (KM) Application Center, Korea Institute of Oriental Medicine, Daegu 41062, Korea; wrld2931@kiom.re.kr; 4 R&D Center, Etnova Therapeutics Corp., 124, Sagimakgol-ro, Jungwon-gu, Seongnam-si 13207, Korea; yhkim@etnova.co.kr; 5 Department of Molecular Medicine, School of Medicine, Kyungpook National University, Daegu 41944, Korea; nams@knu.ac.kr; 6 Department of Chemistry, College of Natural Sciences, Kyungpook National University, Daegu 41566, Korea; ghlee@knu.ac.kr

**Keywords:** glioblastoma (GBM), cancer-derived exosome, anticancer effect, cancer-targeting effect

## Abstract

The use of cancer-derived exosomes has been studied in several cancer types, but the cancer-targeting efficacy of glioma-derived exosomes has not been investigated in depth for malignant glioblastoma (GBM) cells. In this study, exosomes were derived from U87MG human glioblastoma cells, and selumetinib, a new anticancer drug, was loaded into the exosomes. We observed the tropism of GBM-derived exosomes in vitro and in vivo. We found that the tropism of GBM-derived exosomes is in contrast to the behavior of non-exosome-enveloped drugs and non-GBM-specific exosomes in vitro and in vivo in an animal GBM model. We found that the tropism exhibited by GBM-derived exosomes can be utilized to shuttle selumetinib, with no specific targeting moiety, to GBM tumor sites. Therefore, our findings indicated that GBM-derived exosomes loaded with selumetinib had a specific antitumor effect on U87MG cells and were non-toxic to normal brain cells. These exosomes offer improved therapeutic prospects for glioblastoma therapy.

## 1. Introduction

Exosomes are nano-sized extracellular vesicles with a bilayer membrane [1,2]. They play a vital role in multiple normal physiological and pathological processes, acting as communication mediators between cells [3,4]. Exosomes can act as nanocarriers, releasing substances such as proteins and ribonucleic acids (RNAs) into target cells. They can deliver both hydrophilic and hydrophobic molecules and can efficiently target cancer sites [5,6,7]. Among the nanocarriers proposed as valuable for cancer therapy and diagnosis, exosomes have recently received the most attention as promising drug delivery vesicles that can overcome the shortcomings of artificial nanocarriers. In contrast to artificial nanocarriers, exosomes elicit relatively low immune recognition and have low toxicity because exosomes are membrane vesicles that are naturally released from cells and thus have high biocompatibility [8,9,10].

Nanomedicine has made many important contributions to cancer therapy and diagnosis by improving the loading capacity, biodistribution, and target accumulation of therapeutic molecules [11]. Advances in the field of image-guided cancer treatment have been applied to exosomes through the encapsulation of therapeutic molecules and the modification of exosomal membranes to facilitate imaging [12,13]. As a nanoplatform, the inner space of exosomes can be used for the loading of therapeutic molecules. For cancer therapy, many studies have reported the loading into exosomes of a variety of therapeutic drugs, including anticancer drugs, proteins, and nucleic acids [14,15,16]. Exosomes, therefore, appear to offer considerable promise as drug delivery vehicles for cancer imaging and therapy.

Artificial nanoplatforms have been engineered to use various approaches to target cancers. For example, surface modifications of artificial nanoplatforms with cancer cell membranes have been used to achieve the highly specific targeting of drug delivery to tumors and the efficient entry of drugs into cancer cells [17,18]. Cancer-derived exosomes have similar biological contents to their parent cancer cells, so these exosomes might be uniquely capable of communicating with their parent cancer cells [19,20]. Therefore, cancer cell-derived exosomes might be able to return to the parent cancer cells that produced them. This inherent targeting ability of cancer-derived exosomes makes these exosomes better anticancer drug delivery vehicles than other artificial nanoplatforms for the delivery of drugs to cancer sites. Recently, cancer therapy using cancer-derived exosomes has been studied in cancer types, including fibrosarcoma [21,22,23]. Additionally, several studies have reported on the cancer-targeting efficacy of glioma-derived exosomes from glioblastoma (GBM) cells [24,25,26,27,28]. Since GBM is the most common tumor of the brain and has a poor prognosis with high mortality [29], the targeting ability of GBM-derived exosomes is important because GBM involves a complex cancer microenvironment that is composed of both cancerous and non-cancerous cells, including endothelial cells, immune cells, and glial cells [30,31]. The complexity of the GBM microenvironment is further enhanced by hallmark features such as hypoxia [32].

In this study, we isolated exosomes from human glioblastoma cells (U87MG) and loaded selumetinib into the exosomes. Selumetinib is a new anticancer drug used for the treatment of neurofibromatosis type 1 (NF type 1)-related plexiform neurofibromas. Because NF type 1 is highly mutated in GBM, we introduced selumetinib as a good candidate for the treatment of GBM. Furthermore, we hypothesized that the GBM-derived exosomes can be utilized to shuttle selumetinib with no specific targeting moiety to GBM tumor sites with the possible tropism of the exosomes. To investigate the tropism of the exosomes, we compared the therapeutic effects of GBM-derived exosomes with non-GBM-derived exosomes.

## 2. Materials and Methods

### 2.1. Experimental Design

**Figure 1:**
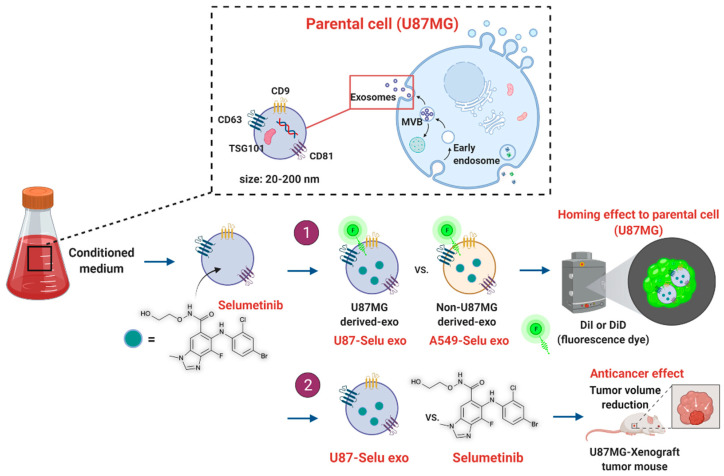
Experimental design of anticancer drug-loaded U87MG exosome.

We isolated the U87MG glioblastoma-derived exosome (U87 exo) from cell media and loaded a novel anticancer drug (selumetinib). Firstly, in vitro and in vivo targeting effect of the drug-loaded U87-exo (U87-Selu exo) into the parental cells was confirmed and compared with the non-U87MG-derived exosome (A549-Selu exo) by fluorescence imaging. The in vitro cancer cell selectivity and anticancer effect of the U87-Selu exo were assessed by measuring cell viability, and native selumetinib was used for comparison to get better insight into the exosome’s drug delivery property. The assessment of the in vivo anticancer effect of U87-Selu exo was performed with an U87MG xenograft mouse model by repeated administration for 10 days. Furthermore, its therapeutic effect and the mechanism of action were compared with native selumetinib by Western blot or flow cytometry (Figure 1).

### 2.2. Cell Culture, Exosome Isolation, Labeling, and Purification

**Figure 2:**
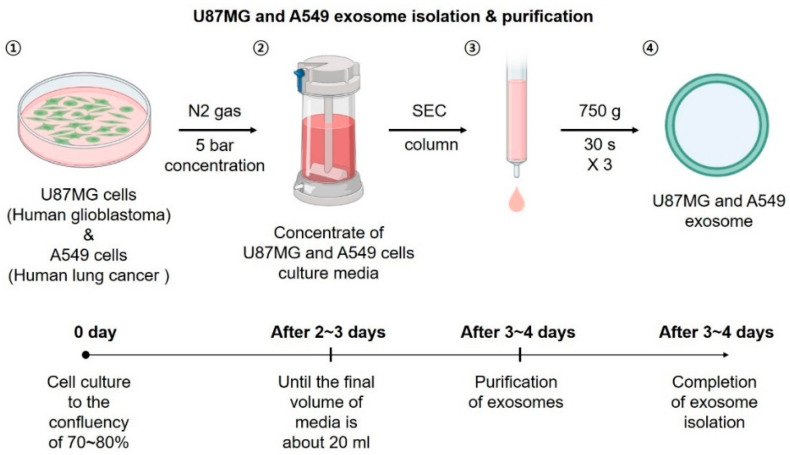
Scheme and time course of exosome isolation.

Human glioblastoma cells (U87MG, ATCC HTB-14), human lung cancer cells (A549, ATCC CCL-185), and normal mouse astrocyte (C8-D1A, ATCC CRL-2541) were purchased from the American Type Culture Collection (ATCC, Manassas, VA, USA). U87MG cells were maintained in Eagle’s minimum essential medium (EMEM; ATCC) supplemented with 10% (*v*/*v*) fetal bovine serum (FBS) and 1% penicillin-streptomycin (PS, Gibco, Thermo Fisher Scientific, Waltham, MA, USA catalog no. 11548876). A549 cells were maintained in RPMI 1640 (Roswell Park Memorial Institute Medium; WelGENE, Daegu, Korea) supplemented with 10% FBS (Gibco) and 1% PS. C8-D1A cells were maintained in Dulbecco’s modified Eagle’s medium (DMEM, WelGENE, Cat.no. LM001-05) supplemented with 10% FBS and 1% PS. Cells were maintained at 37 °C in a humidified incubator under an atmosphere of 5% CO_2_. When the cells reached 70–80% confluence, they were conditioned with 10% exosome-depleted FBS (System Biosciences Inc., Palo Alto, CA, USA) to serum starvation and 1% PS to isolate exosomes. After 48–72 h, the cell medium was collected and centrifuged at 3000× *g* for 15 min to remove cellular debris. The supernatant was then transferred to a new tube and condensed using a 50 mL Amicon Stirred Cell (UFSC05001; Millipore, Burlington, MA, USA) and a Biomax 30 kDa Ultrafiltration Membrane (PBTK04310; Millipore). Exosomes were isolated using ExoQuick-TC ULTRA EV Isolation Kits for Tissue Culture Media (EQULTRA-20TC-1, System Biosciences Inc.) according to the manufacturer’s instructions (Figure 2) [33,34].

To track the exosomes, we labeled the exosomes with fluorescent dye using Vybrant Multicolor Cell-Labeling Kits (V22889, Thermo Fisher Scientific) and an Exosome Spin Column (MW3000) (4484449, Invitrogen, Carlsbad, CA, USA) was used to remove the free fluorescent dye.

### 2.3. Fabrication and Purification of Selu-Exo

Selumetinib (ChemScene, Monmouth Junction, NJ, USA, Cat. no. CS-0059, Lot. 78580)-loaded U87 MG-derived exosomes (U87-Selu exo) were fabricated by the electroporation method (4D Nucleofector; Lonza, Basel, Switzerland) using an Amaxa SE Cell Line 4D-NucleofectorTM X Kit L (24RCT, Catalog No. V4XC-1024; Lonza) according to the manufacturer’s instructions. The pulsation program for encapsulation was DS-126. The excess free selumetinib was removed by filtering through an Amicon Ultra 0.5 mL Centrifugal Filter (3K, Catalog No. UFC500396; Millipore) and washed twice with phosphate buffered saline (PBS) at pH 7.4 (1X, Catalog No. 10010-023, Gibco). For selumetinib loading into A549-derived exosomes (A549-Selu exo), Amaxa SF Cell Line 4D-NucleofectorTM X Kits L (24RCT, Catalog No. V4XC-2024; Lonza) and the CM-130 pulsation program were used. Further purification of A549-Selu exo was conducted with the same preparative method as for U87-Selu exo. The amount of selumetinib was determined by a UV-vis spectrophotometer (DS-11; DeNovix, Wilmington, DE, USA) at an absorbance wavelength of 265 nm (Supplementary Materials, Figure S1). Additionally, the selumetinib loading percentage (*w*/*w*%) was calculated as follows: selumetinib loading percentage (*w*/*w*%) = (encapsulated selu amount/total selu amount) × 100. The experiment was performed in triplicate for independent batches.

### 2.4. Characterization of Exosomes

#### 2.4.1. Size Distribution Analysis

To quantify the purified exosome, the exosome solution (5 mg/mL sterilized PBS, pH 7.4, 10010-023, Gibco) was filtered through a cellulose syringe filter with a 0.20 µM pore size (13CP020AS, ADVANTEC, Dublin, CA, USA). The diameter and concentration of the exosomes were determined with an NTA system (Nanosight NS 300, Malvern Panalytical, Malvern, UK), and imaging parameters were as follows: camera level = 14; screen gain = 3.0; detection threshold = 3; the number of frames = 1498. All size distribution analysis was performed in triplicate for independent batches.

#### 2.4.2. Transmission Electron Microscope (TEM)

A total of 10 μL exosome solution (5 μg/mL, in PBS) was put on a copper grid for 10 min. Then, the same grid was put on a uranyl acetate solution (2% *w*/*v*, 10 μL) to stain the sample for 10 min. The copper grid was photographed using transmission electron microscopy (TEM), (JEM-2200FS, JEOL, Tokyo, Japan) at 200 keV.

#### 2.4.3. Western Blot Analysis

Protein expression was assessed using Western blot analysis. Protein was extracted in radioimmunoprecipitation assay lysis buffer (Millipore) containing a protease and phosphatase inhibitor cocktail (Roche Diagnostics, Basel, Switzerland). Protein lysates (35 µg exosome) were separated using electrophoresis in sodium dodecyl sulfate polyacrylamide gels, and the separated proteins were electrotransferred onto PVDF membranes. The membranes were blocked in a 3% bovine serum albumin (BSA) Tris-buffered saline solution containing 0.05% Tween 20 (TBS-T) for 1 h at room temperature and then incubated with the following diluted primary antibodies: CD9 recombinant rabbit m antibody (1:1000 dilution, Invitrogen, Catalog No. MA5-31980), CD63 polyclonal antibody (1:1000 dilution, Invitrogen, Catalog No. PA5-100713), CD81 recombinant rabbit monoclonal antibody (1:1000 dilution, Invitrogen, Catalog No. MA5-32333), TSG101 monoclonal antibody (1:1000 dilution, Invitrogen, Catalog No. MA1-23296), or Calnexin polyclonal antibody (1:2000 dilution, Invitrogen, Catalog No. PA5-34754) in 3% BSA for 3 h at room temperature. The membrane was then incubated with horseradish-conjugated secondary antibody (1:2000 dilution, Cell Signaling Technology, Danvers, MA, USA) for 2 h at room temperature. After washing with TBS-T, immunoreactive bands were visualized using the Chemiluminescence Western Imaging System (Supernova-Q1800TM; Centronics, Daejeon, Korea). Immunoblotting analysis was performed in triplicate for independent batches.

#### 2.4.4. Stability of Selumetinib-Loaded Exosomes

The stability of the U87 or A549-Selu exo samples (2.5 μg/μL in sterilized PBS) was checked by monitoring size distribution changes with a Nanoparticle Tracking Analysis (NTA) system (camera level: 14, screen gain: 3.0, detection threshold: 3) at a physiological pH of 7.4 for 7 days. The stability assessment was performed in triplicate for independent batches.

### 2.5. In Vitro Exo and Selu-Exo Internalization to the Parent Cell

To track the exosomes, we labeled the U87-exo and U87-Selu exo with a fluorescent dye (1, 1′-Dioctadecyl-3, 3, 3′, 3′-tetramethylindocarbocyanine perchlorate, DiI, V22885, Thermo Fisher Scientific) at 37 °C for 1 h [35,36,37]. The fluorescence intensities of DiI-labeled exosomes (DiI-U87-exo and DiI-U87-Selu exo) were compared with 10 μL of diluted DiI solution (0.02 μL stock/μL methanol) at 530 nm excitation and 620 nm emission with a microplate reader (SpectraMax^®^ i3, Molecular Devices, San Jose, CA, USA) (Supplementary Materials, Figure S2).

For internalization images of exosomes to parental cells, U87MG cells were seeded in 4-well cell culture slides at 4 × 10^4^ cells/well (SPL Life Sciences, Pocheon, Korea). The following day, they were treated with 650 µg/µL of DiI-labeled exosomes (DiI-U87-exo and DiI-U87-Selu exo). Non-treated cells were used as a control. After incubation for 12 h at 37 °C, the cells were washed with Dulbecco’s phosphate-buffered saline (DPBS, WelGENE, Daegu, Korea, Catalog No. LB 001-01). The chambered slides were fixed with 4% paraformaldehyde (PFA) for 15 min and then washed with TBS. They were mounted with VECTASHIELD Antifade Mounting Medium with 4′,6-diamidino-2-phenylindole (DAPI) (Vector Laboratories, Burlingame, CA, USA; Catalog No. H-1200). The slides were captured using a Nikon fluorescence microscope and the software NIS-Elements BR 5.11 (Nikon, Tokyo, Japan). The imaging was performed in triplicate. Additionally, to quantify the uptake extent of the exosomes to parental cells, U87MG cells were seeded in 96-well plates at 2 × 10^4^ cells/well (SPL Life Sciences). The following day, the cells were treated with 300 μg/uL of DiI-labelled exosomes (DiI-U87 exo and DiI-U87-Selu exo), and the control cells were not treated. After incubation for 12 h at 37 °C, the fluorescence intensity was measured using a microplate reader (SpectraMax^®^ i3; Molecular Devices, ex: 530 nm; em: 620 nm). For A549 cells as a negative control, the same method was used. The quantification experiment was performed 8 times repeatedly.

### 2.6. Cell Viability Treated with Exosome, Selumetinib, and U87-Selu Exo 

Cell viability was detected using the Cell Counting Kit-8 (D-Plus^TM^ CCK cell viability assay kit, Dongin LS, Korea). U87MG, A549 and C8-D1A cells were seeded in 96-well plates (SPL Life Sciences) at 1 × 10^4^ cells/well. The following day, the cells were treated with exosome (0, 10, 20, 40, 60, 80 and 100 μg/μL), selumetinib (0, 10, 20, 40, 60, 80 and 100 μg/μL) and Selu-exo (0, 10, 20, 40, 60, 80 and 100 μg/μL) in EMEM, RPMI 1640 and DMEM serum-free media, respectively. After 24 h, CCK-8 solution (10 μL) was added to each well, and the plate was incubated for 2 h at 37 °C, 5% CO_2_ atmosphere. The absorbance of the cell culture plate was read at 450 nm using a microplate reader. All experiments were independently performed 3 times. The graph of log IC_50_ values represents average values.

### 2.7. Animals

For all experiments, a male Balb/c-nude mouse (Nara Biotech, Seoul, Korea) weighing 16–18 g was used. Animals were housed in controlled environmental conditions with a light-dark cycle of 12 h at an ambient temperature of 23 ± 1 °C and relative humidity of 50 ± 10% and had free access to food and water. All animal experiments were approved and performed according to the guidelines of Kyungpook National University Animal Research Center (IACUC) (No. KNU-2021-0225; Daegu, Korea, 24 December 2021).

### 2.8. U87MG Xenograft Model

For the generation of a tumor xenograft, U87 MG cells (3 × 10^6^ cells/100 µL EMEM medium, serum-free) were subcutaneously injected into the upper-left flank region of the male Balb/c-nude mice. Then, the mice recovered on the 37 °C warm pad. When the tumor reached a mean size of about 100 mm^3^, mice were randomized into each experimental group: saline (*n* = 6), U87-Selu exo (*n* = 6), and A549-Selu-exo (*n* = 5).

### 2.9. In Vivo Exo and Selu-Exo Targeting to the Parental Tumor

**Figure 3:**
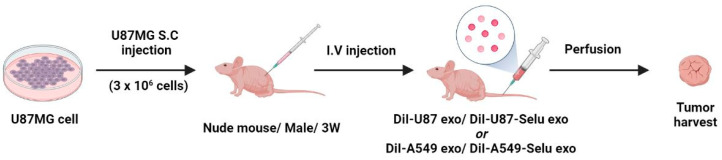
Overview of the DiI-labelled exosomes (DiI-U87 exo, DiI-U87-Selu exo, DiI-A549 exo and DiI-A549-Selu exo) in vivo targeting experiments.

To observe the in vivo targeting effect of exosomes on tumor tissue, DiI-labeled exosomes (DiI-U87 exo, DiI-A549 exo, DiI-U87-Selu exo and DiI-A549-Selu exo; 8–9 µg/µL exosome) were injected intravenously into U87 xenograft mice tail vein with 50 mg exosome/kg dose (*n* = 5 for each group) (Figure 3). A total of 24 h after injection, the mice were sacrificed by exsanguination from the vena cava for tumor harvesting. The harvested tumors were embedded in optical cutting temperature (OCT) compound. The OCT compound sections were then cut into thicknesses of 8 µm. After the specimens were washed 3 times with TBS (100 mL), the slides were covered with VECTASHIELD mounting medium with DAPI (Vector Laboratories; Catalog No. H-1200). Images of the slide sections were captured using a Nikon fluorescence microscope with the software NIS-Elements BR 5.11 (Nikon). The relative fluorescence intensity was analyzed by ImageJ software (Version 1.50i; US National Institutes of Health, Bethesda, MD, USA).

### 2.10. In Vivo Biodistribution of U87-Selu Exo

To observe the in vivo biodistribution of U87-Selu exo, fluorescent dye (1,1′-dioctadecyl-3,3,3′,3′-tetramethylindodicarbocyanine perchlorate; DiD)-labeled U87-Selu exo (DiD-U87-Selu exo, 8–9 µg/µL exosome) was intravenously injected into the U87MG tumor xenograft mice tail vein with 10 mg exosome/kg dose (*n* = 3). A total of 24 h after injection, the mice were sacrificed by exsanguination from the vena cava. The tumor, liver, kidney, lung, heart, spleen, and plasma were harvested to quantify the relative fluorescence intensity induced by the accumulated DiD-U87-Selu exo. The harvested organs were imaged using an IVIS Spectrum imaging system (Lumina 3, PerkinElmer, Waltham, MA, USA).

### 2.11. In Vivo Anticancer Effect of U87-Selu Exo and A549-Selu Exo

**Figure 4:**
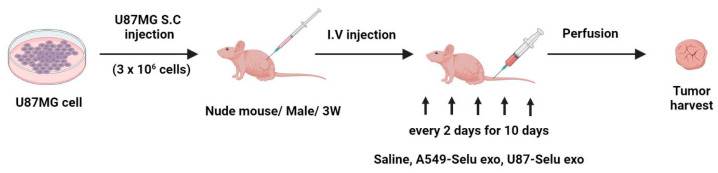
Overview of in vivo anticancer effect of U87-Selu exo and A549-Selu exo using a U87MG xenograft model.

U87-Selu exo (*n* = 6), A549-Selu exo (*n* = 6), or saline (*n* = 5) were intravenously injected into U87MG xenograft mice tail vein once every 2 days for 10 days (Figure 4). After injection, the mice recovered on the 37 °C warm pad. The injected volume was 100 µL for each of the three groups, and the amount of selumetinib loaded was 1 mg for U87-Selu exo and A549-Selu exo (8–9 µg/µL exosome). The tumor size was checked using a digital caliper every 2 days, and the tumor volume was calculated with the following formula: tumor volume = length × (width)^2^ × 0.5. The anticancer effect was expressed as a percentage of tumor growth inhibition (% TGI) calculated using the following equation: 100 − (T/C × 100), where T is the mean relative tumor volume (RTV) of treated tumors and C is the mean RTV of the saline control group at the time of sacrifice. RTV = Vx/V1, where Vx is the volume in mm^3^ at a given time and V1 is the start of treatment. Mean TGI (%) and standard deviation were calculated for each group.

### 2.12. Histological Analysis for Tumor Tissue

After five intravenous injections, the mice were dissected and perfused. The tumor was harvested from each mouse, and the harvested tumors were fixed with 4% PFA for 3 days. Fixed tumors were treated with an alcohol concentration gradient (50, 70, 95, and 100%), xylene (Junsei Chemical, Tokyo, Japan), and paraffin for 30 min, respectively. The paraffin-embedded tumor tissues were sectioned (5 μm) and subjected to immunofluorescence (IF) and immnunohistochemical (IHC) staining by treatment with xylene and alcohol concentration gradients (100, 95, 70, and 50%) in an oven at 65 °C for 1 h and 10 min, respectively.

For IHC staining, endogenous peroxidase activity was inactivated by incubation in 0.3% H_2_O_2_ in methanol (Sigma-Aldrich, St. Louis, MO, USA) for 10 min. The sections were then rinsed with 0.1 M TBS (pH 7.4) and boiled in citrate buffer (pH 6.0) containing 0.03% Tween-20 for 4 min. Finally, the sections were incubated with a blocking solution (5% normal goat serum (NGS) and BSA in TBS) at 25 °C for 1 h, and indirect immunization occurred with an antibody to anti-Ki-67 (diluted 1:100) for 1 h applied to the histochemistry. For negative controls, the primary antibody was omitted, and slides were incubated with a blocking solution. Sections were then incubated with horseradish peroxidase (HRP)-conjugated anti-rabbit IgG for 1 h at 25 °C, stained with VECTOR1 NovaRED (Vector Laboratories, Inc.), and counterstained with hematoxylin (BBC Biochemical, Mount Vernon, WA, USA). Sections were dehydrated, cleaned, and mounted with Permount (Fisher, Fair Lawn, NJ, USA). Images were captured with a fluorescence microscope (ECLIPSE Ti, Nikon), and the fluorescence intensity of immunostaining was quantified using ImageJ.

For IF staining, specimens were blocked with TBS supplemented with 5% NGS and 5% BSA for 2 h and incubated overnight with primary antibody (anti-cleaved-caspase-3, diluted 1:100) in blocking solution (5% NGS and BSA) at 4 °C. Sections were then washed three times in TBS and incubated for 1 h in the presence of Alexa Fluor conjugated IgG labeled secondary antibody (Invitrogen, Carlsbad, CA, USA). Sections were washed and mounted with Vectashield mounting medium containing DAPI (Vec-tashield H-1500; Vector Laboratories, Inc., Burlingame, CA, USA). Fluorescence microscopy (ECLIPSE Ti, Nikon) was used to capture images, and ImageJ was used to quantify the fluorescence intensity of immunostaining.

### 2.13. In Vivo Toxicity of U87-Selu Exo and A549-Selu Exo

To evaluate in vivo toxicity of U87-Selu exo and A549-Selu exo, the bodyweight of the mice (*n* = 5 for each group) used for the assessment of the anticancer effect was measured using an electronic scale at the end of the dosage.

Additionally, glutamate oxaloacetate transaminase (GOT) and glutamic-pyruvic transaminase (GPT) levels were measured to evaluate the liver function of the mice. For the GOT and GPT measurements, we extracted blood (500 μL) from the mice’s abdominal vena cava. The extracted blood was incubated at room temperature for 2 h and then centrifuged at 2000× *g* for 15 min. Finally, the supernatant serum (500 μL) was used for GOT and GPT measurements.

To evaluate the histological toxicity, the liver and kidney were collected and fixed with 4% paraformaldehyde buffer over 3 days. After washing and dehydrating the tissues, sections were made by paraffin embedding and cut into 5 µm slices. Finally, the sections were stained with hematoxylin and eosin (H&E) following the protocol using Eosine Y Alcoholic (BBC Biochemical Corp., Mount Vernon, WA, USA; catalog No. 3605). Some sections were stained using Picro Sirius Red stain kits (Abcam, Cambridge, UK; Catalog No. ab150681).

### 2.14. In Vitro Anticancer Effect of U87-Selu Exo

U87MG cells were seeded onto a 60 mm^2^ cell culture plate (8 × 10^5^ cells/plate, SPL Life Sciences). After 12, 24, or 48 h, they were treated with 100 µg/µL of U87-Selu exo and selumetinib. The medium was discarded, and the cells were washed with DPBS. The extracted protein lysates (10 µg/µL) that were used to determine protein concentration by a BCA assay were separated via electrophoresis on sodium dodecyl sulfate-polyacrylamide gels, and the separated proteins were electrotransferred onto PVDF membranes. Membranes were incubated with the following diluted primary antibodies: β-actin (1:2000, Santa Cruz Biotechnology, Dallas, TX, USA; Catalog No. sc-47778), p38 (1:1000, Cell Signaling Technology; Catalog No. 9212), p-p38 (1:1000, Cell Signaling Technology; Catalog No. 9215), p27 (1:1000, Cell Signaling Technology; Catalog No. 3686), poly(ADP-ribose) polymerase (PARP) (1:1000, Cell Signaling Technology; Catalog No. 9532), Bax (1:1000, Abcam; Catalog No. ab32503), Bcl2 (1:1000, Abcam; Catalog No. ab59348), proliferating cell nuclear antigen (PCNA, 1:1000, Cell Signaling Technology; Catalog No. 2586),Cyclin D1 (1:1000, Cell Signaling Technology; Catalog No. 55506), ERK (1:1000, Cell Signaling Technology; Catalog No. 9102), protein kinase-like endoplasmic reticulum kinase (pERK, 1:1000, Cell Signaling Technology; Catalog No. 3101), mitogen-activated protein kinase (MEK) 1/2 (1:1000, Cell Signaling Technology; Catalog No. 9122), phospho-mitogen-activated protein kinase (pMEK) 1/2 (1:1000, Cell Signaling Technology; Catalog No. 9121), Ras (1:1000, Cell Signaling Technology; Catalog No. 3965), c-Raf (1:1000, Cell Signaling Technology; Catalog No. 9422), or p-c-Raf (1:1000, Cell Signaling Technology; Catalog No. 9421) in 3% BSA for 3 h at room temperature. The membrane was then incubated with horseradish-conjugated secondary antibody (1:2000, Cell Signaling Technologies) for 2 h at room temperature. After washing with TBS-T, immunoreactive bands were visualized using the Chemiluminescence Western Imaging System (Supernova-Q1800TM; Centronics, Daejeon, Korea). All Western blotting was performed in triplicate.

### 2.15. Flow Cytometry Analysis

U87MG cells were seeded on a 60 mm^3^ cell culture dish (8 × 10^5^ cells/dish, SPL Life Sciences). After 24 h, the cells were treated with 200 µg/µL of native U87MG cell-derived exosome (exo), U87-Selu exo and selumetinib for 24 h. The medium was discarded, and the cells were washed with DPBS. Trypsin-2,2′,2″,2′″-(Ethane-1,2-diyldinitrilo)tetraacetic acid (EDTA) was added, and the cells were collected in DPBS + 2% FBS. The cells were centrifuged at 4000 rpm for 3 min, washed, and resuspended in DPBS + 2% FBS. The cells were fixed with 100% ethanol and incubated overnight at 4 °C. The next day, they were centrifuged, the ethanol was discarded, and the cells were washed with DPBS + 2% FBS. The pellet was resuspended in 1.12% sodium citrate buffer (pH 8.4) with 50 µg/mL RNase and incubated at 37 °C for 30 min. Finally, propidium iodide (PI) working solution (50 µg/mL) was added and incubated for 30 min at room temperature. The PI (50 µg/mL, propidium iodide, Invitrogen, Catalog No. P21493)-stained cells were analyzed using a CytoFLEX Flow Cytometer (Beckman Coulter Life Science, Brea, CA, USA) to determine the relative DNA content based on the red fluorescence. The experiment was performed in triplicate.

### 2.16. Statistical Analysis

Statistical analysis was done using one-way ANOVA followed by the Dunnett comparison test also using two-way ANOVA followed by Bonferroni post-tests to compare replicate means by row. Values of *p* < 0.05, *p* < 0.01 and *p* < 0.001 are represented by *, ** and *** vs. nontreated control and #, ##, and ### vs. treated control, respectively. GraphPad Prism (version 5.02; GraphPad Prism Software Inc., SanDiego, CA, USA) and Excel programs were used to draw figures and graphs.

## 3. Results and Discussion

### 3.1. Exosome Characterization

**Figure 5:**
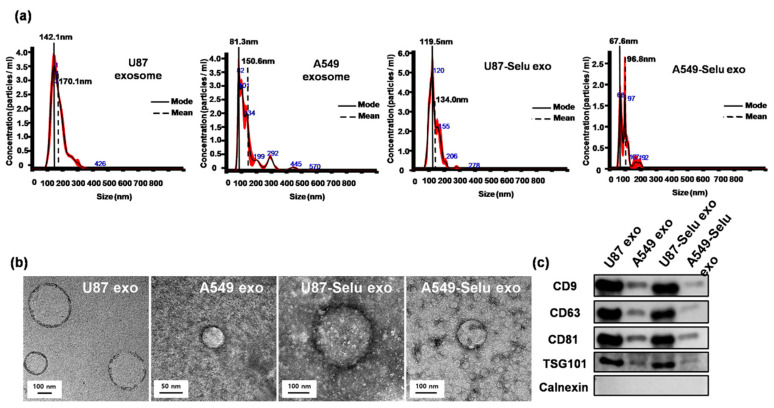
Characterization of U87 exo, A549 exo, U87-Selu exo, and A549-Selu exo. (**a**) NTA results showed the mean and mode size of the exosomes (black: mean value, red: error). The loading of selumetinib changed the size distribution of the exosomes for both U87 and A549 derived exosomes. *n* = 3. (**b**) TEM images showed that all exosomes were round in shape. (**c**) Immunoblotting results revealed that U87-derived exosome-specific exosomal markers (CD proteins and TSG 101) were highly expressed in U87-derived exosomes, but not in A549-derived exosomes. A non-exosomal marker (Calnexin) was used as reference, *n* = 3.

The mean size of exosomes from the NTA and TEM measurements was 170.0 ± 3.3 nm for U87 exosomes, 150.6 ± 2.5 nm for A549 exosomes, 134.0 ± 0.8 nm for U87-Selu exosomes, and 96.8 ± 4.6 nm for A549-Selu exosomes (Figure 5a,b). The average size and the distribution slightly differed after selumetinib loading into each native exosome, but the morphology was maintained as a round shape. The Western blot data also showed that the exosome-specific protein markers CD9, CD63, and CD81 (endosome-specific tetraspanins) and TSG101 (an exosome biogenesis protein) were not changed by loading selumetinib into the exosomes (Figure 5c). These data indicated that the exosomes were successfully isolated from the cell culture media. The selumetinib loading efficiency (%) was calculated using a UV-vis spectrometer to be 80–90% for the U87 exo and A549 exo. U87-Selu exo showed size stability for up to 7 days at pH 7.4 (Supplementary Materials, Figure S3).

### 3.2. U87MG Exosome Targeting In Vitro and In Vivo

**Figure 6:**
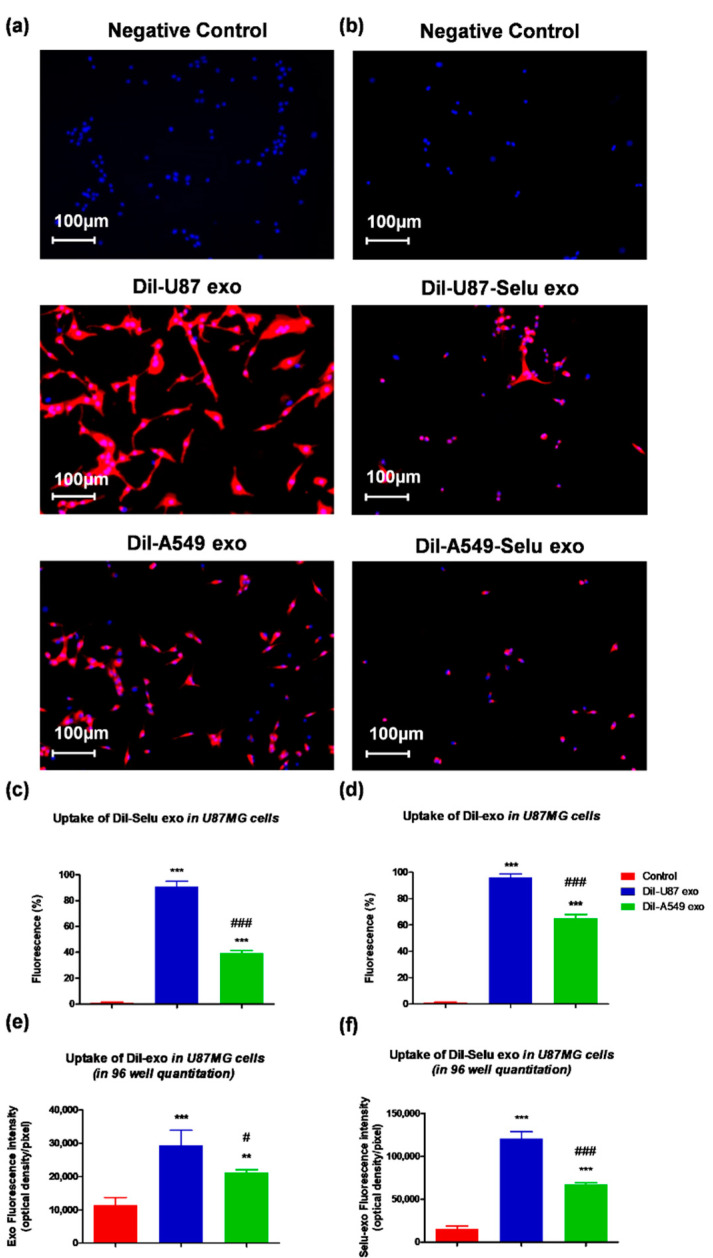
In vitro exosome targeting to U87 cells. (**a**,**c**) DiI-U87 exo showed higher fluorescence intensity than DiI-A549 exo in U87 cells. *** *p* < 0.001 vs. non-treated control, ### *p* < 0.001 vs. DiI-A549-Selu exo. *n* = 3. (**b**,**d**) DiI-U87-Selu exo showed higher fluorescence intensity than DiI-A549-Selu exo in U87 cells. *** *p* < 0.001 vs. control, ### *p* < 0.001 vs. DiI-A549-Selu exo. *n* = 3. (**e**,**f**) In the 96-well plate, the quantitative uptake of DiI-U87 exo and DiI-U87-Selu exo were higher than DiI-A549 exo and DiI-A549-Selu exo. ** *p* < 0.01, *** *p* < 0.001 vs. non-treated control; # *p* < 0.05, ### *p* < 0.001 vs. DiI-A549-Selu exo. *n* = 8. Data are represented as mean ± SD.

We used the fluorescence dye (DiI)-labelled exosomes (DiI-U87 exo and DiI-A549 exo) to observe the targeting effect on bioimaging. As a negative control, non-treated cell stained with DAPI was used for cell localization. In vitro exosome-targeting experiments revealed that DiI-U87 exo showed higher fluorescence intensity than DiI-A549 exo, suggesting 1.4-fold more uptake of DiI-U87 exo than DiI-A549 exo by U87MG cells (Figure 6a,c). As a negative control, DAPI staining was performed without the fluorescent dye DiI. DiI-U87-Selu exo, which was loaded with selumetinib, also showed higher fluorescence intensity than DiI-A549-Selu exo, suggesting 1.8-fold more uptake of DiI-U87-Selu exo than of DiI-A549-Selu exo by U87MG cells (Figure 6b,d). Additionally, to quantitatively compare the uptake of exosomes and selumetinib-loaded exosomes, we observed another targeting experiment with 96-well cell culture plates. As a result, the uptake of U87 exo was 1.5-fold higher than A549 exo, and U87-Selu exo was 2.3-fold higher than A549-Selu exo (Figure 6e,f). These results, therefore, indicated that U87-exo is more efficiently taken up by their parent U87MG cell lines than by A549-exo. These in vitro results also demonstrated that the loading of selumetinib into exosomes did not much change the targeting efficiency of the exosomes to their parent cell lines.

**Figure 7:**
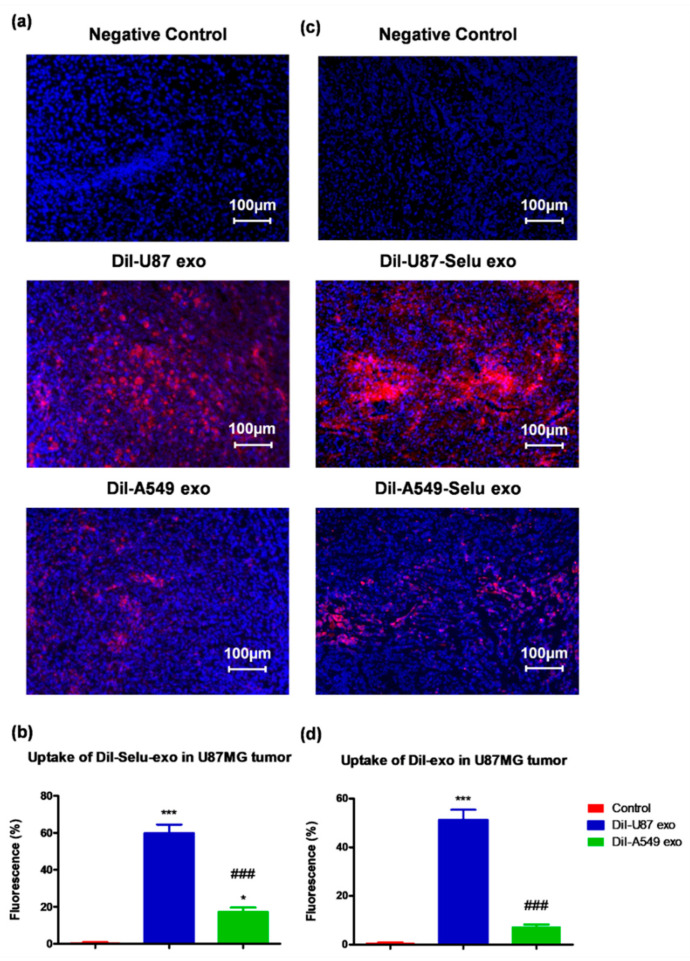
In vivo exosome targeting cancer tissue of U87 cells using a U87MG glioblastoma xenograft mice. (**a**,**b**) DiI-U87 exo showed higher fluorescence intensity than DiI-A549 exo in U87 cells. * *p* < 0.05, *** *p* < 0.001 vs. non-treated control, ### *p* < 0.001 vs. DiI-A549-Selu exo, *n* = 5. (**c**,**d**) DiI-U87-Selu exo showed higher fluorescence intensity than DiI-A549-Selu exo in U87 cells. *** *p* < 0.001 vs. non-treated control, ### *p* < 0.001 vs. DiI-A549-Selu exo, *n* = 5. Data are represented as mean ± SD.

In order to investigate whether exosomes were targeting their parent cells in vivo, DiI-U87 exo and DiI-A549 exo were intravenously injected into U87MG cell-implanted GBM xenograft mice. Non-treated negative controls did not represent any fluorescence without DAPI. DiI-U87 exo showed 6.9-folds much higher fluorescence intensity than DiI-A549 exo, suggesting more uptake of DiI-U87 exo than of DiI-A549 exo by GBM tumors (Figure 7a,b). In the case of selumetinib-loaded exosomes, DiI-U87-Selu exo showed 3.5-fold higher fluorescence intensity than DiI-A549-Selu exo, suggesting more uptake of DiI-U87-Selu exo than of DiI-A549-Selu exo by GBM tumors (Figure 7c,d). Compared with the in vitro results, the in vivo results revealed that the difference in uptake was larger between DiI-U87 exo and DiI-A549 exo and between DiI-U87-Selu exo and DiI-A549-Selu exo. Because the in vivo case is more realistic than the in vitro case for the evaluation of the targeting effect to parent cancer cells, the results from the in vitro and in vivo experiments strongly suggest that U87-derived exosomes have higher targeting efficacy than A549-derived exosomes to GBM tumors from parent U87MG cells. This high targeting efficacy of U87-derived exosomes was not changed by loading an anticancer drug, even in vivo. Although the in vivo ability of cancer cell-derived exosomes to return to the parent cancer cells is often called the “homing effect” [19,20], it is not appropriate to describe our in vivo result as a homing effect due to the ectopic tumor model. To solve this limitation, further study is warranted to investigate the possible homing effect using an appropriate tumor model.

### 3.3. Cytotoxicity of U87 Exo, Selumetinib and U87-Selu Exo

**Figure 8:**
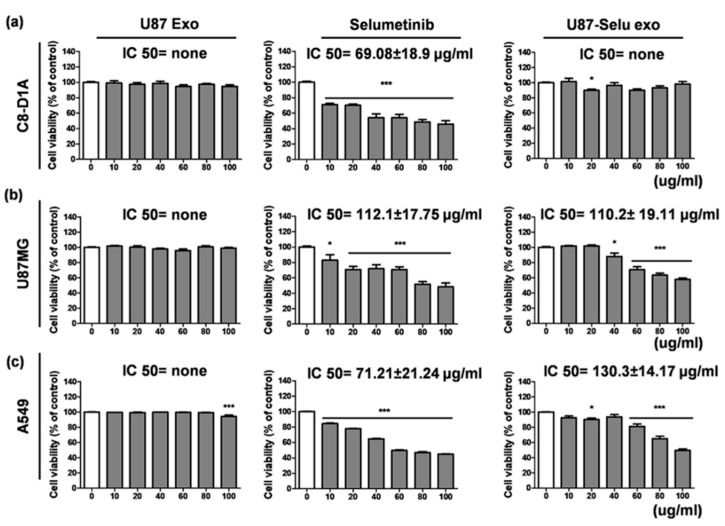
Cytotoxicity of U87 exo, selumetinib, and U87-Selu exo of (**a**) C8-D1A (mouse normal brain cells), (**b**) U87MG (human glioblastoma cells), and (**c**) A549 (human lung cancer cells) cells at various concentrations. * *p* < 0.05, *** *p* < 0.001 vs. non-treated viability value of each data, *n* = 3. Data represented as mean ± SD.

To investigate the cytotoxicity of the U87 exo, we conducted cell viability experiments using U87MG cells, A549 cells, and C8-D1A cells. Native U87 exo represented by the values of IC50 were none in all cell lines (Figure 8a). This result suggests that U87 exo, which were cancer-derived exosomes, did not have cytotoxic effects on either normal or cancer cells. However, selumetinib inhibited cellular proliferation in the same condition, representing values of IC50 = 69.08 ± 18.9 µg/mL for C8-D1A cells, 112.1 ± 17.75 µg/mL for U87MG cells, and 71.21 ± 21.24 µg/mL for A549 cells (Figure 8b). Even though U87-Selu exo has a relatively similar or low growth inhibition effect to native selumetinib for two cancer cells, it showed proliferation inhibition effects for cancer cells (IC50 value = 110.2 ± 19.11 µg/mL for U87MG and 130.3 ± 14.17 µg/mL for A549) without normal cell toxicity (IC50 value = none for C8-D1A) (Figure 8c). Additionally, the cytotoxic effect of U87-Selu exo was stronger in U87MG cells than in A549 cells and may result from the high tumor targeting of U87-Selu exo to its parental cell (U87MG). These results, therefore, suggest that U87-Selu exo had higher anticancer efficiency against U87MG cells than A549 cells.

### 3.4. In Vivo Biodistribution of U87-Selu Exo

**Figure 9:**
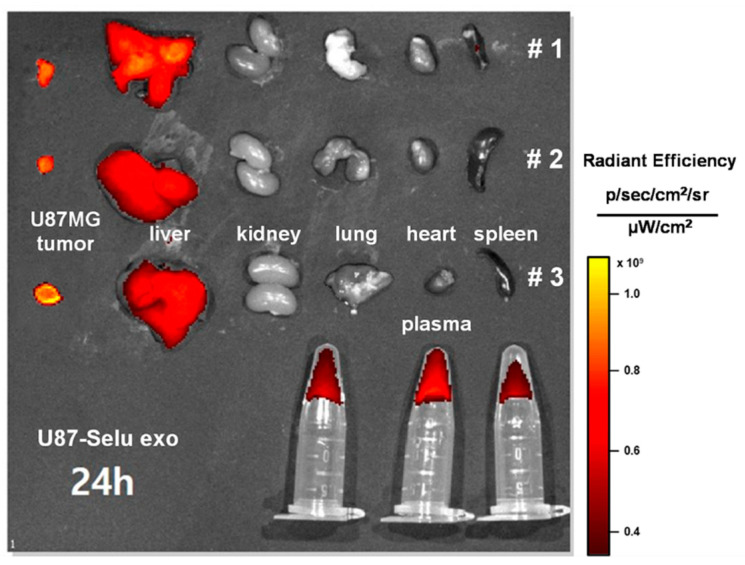
In vivo biodistribution of U87-Selu exo. A strong fluorescence signal was found in the tumor and liver, but not in other organs; # means independent mouse number. *n* = 3.

To investigate the in vivo biodistribution of U87-Selu exo, nude mice bearing U87MG tumors were intravenously injected with DiD-labeled U87-Selu exo (DiD-U87-Selu exo). After 24 h, the mice were sacrificed, and the tumor, liver, kidney, lung, heart, spleen, and plasma were harvested. The organ images were taken using an IVIS fluorescence imaging device. The tumors showed the strongest fluorescence signal, suggesting that they had the highest uptake of U87-Selu exo (Figure 9). A fluorescent signal was also found in the liver but not in other organs. The liver uptake of cancer-derived exosomes has been reported in previous studies [38,39,40]. These previous biodistribution studies of intravenously injected exosomes showed the rapid clearance of exosomes by the liver using a reticuloendothelial system. A fluorescent signal was found in plasma samples, indicating a long circulation of U87-Selu exo in the bloodstream. Therefore, the in vivo biodistribution results demonstrated targeted delivery of U87-Selu exo to tumors due to the targeting effect on parent tumor cells.

### 3.5. Liver and Kidney Toxicity of Selu-Exo (U87-Selu Exo and A549-Selu Exo) In Vivo

**Figure 10:**
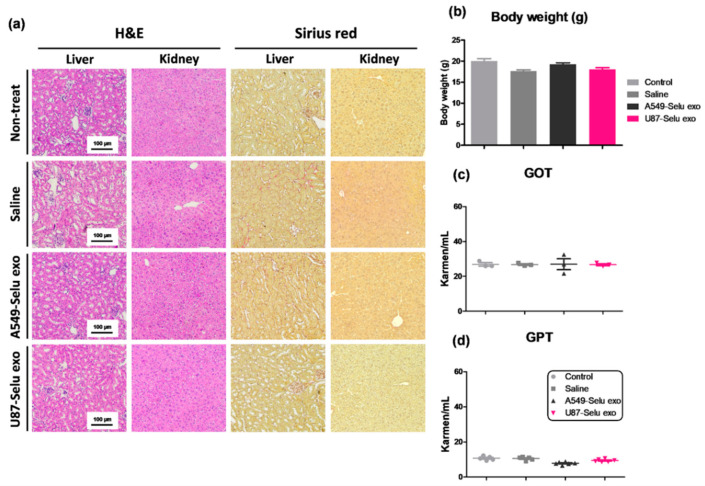
In vivo liver and kidney toxicity of U87-Selu exo and A549-Selu exo. (**a**) Bodyweight of U87MG-bearing mice treated with saline, A549-Selu exo, and U87-Selu exo. *n* = 5. Data represented as mean ± SD. Liver toxicity measurement. (**b**) GOT, (**c**) GPT, and (**d**) hematoxylin and eosin and Sirius red staining of liver and kidney.

Due to the liver uptake of U87-Selu exo, the possible liver toxicity of U87-Selu exo and A549-Selu exo was examined. Hematoxylin and eosin-stained and Sirius red-stained images revealed no pathological changes in the kidney or liver of U87-Selu exo- or A549-Selu exo-treated mice (Figure 10a). Additionally, no change in body weight was observed after U87-Selu exo and A549-Selu exo injection in mice, and the GOT and GPT scores, which reflect liver function, were similar in control mice and saline-injected mice (Figure 10b–d).

### 3.6. In Vivo Anticancer Effects of U87-Selu Exo and A549-Selu Exo

**Figure 11:**
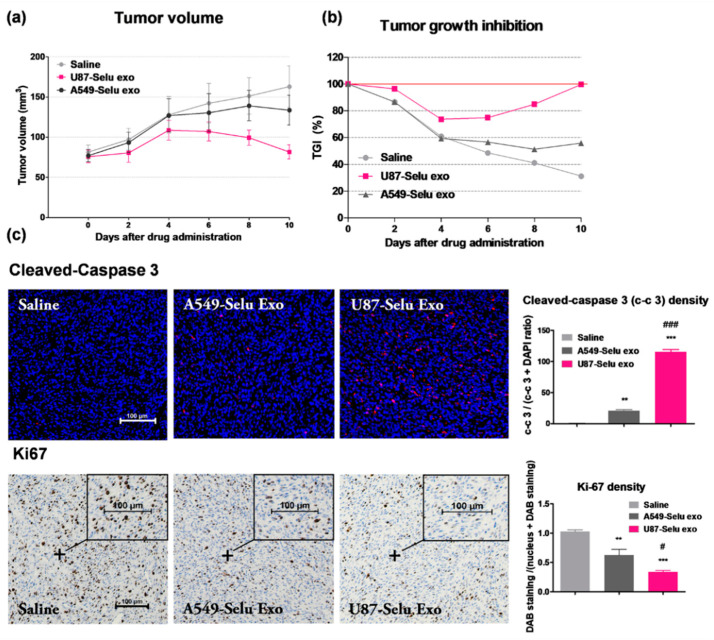
Anticancer effects of U87-Selu exo and A549-Selu exo in vivo. (**a**) Tumor volume changes up to 10 days after tumor induction, *n* = 5. (**b**) Tumor growth inhibition changes up to 10 days after tumor induction (red line: 100 % TGI). (**c**) Cleaved caspase-3 and Ki67 immunohistochemistry images of tumor. ** *p* < 0.01, *** *p* < 0.001 vs. saline group; # *p* < 0.05 and ### *p* < 0.001 vs. A549-Selu exo, *n* = 3. Scale bar: 100 µm. Data represented as mean ± SD.

To evaluate the anticancer effect of two selumetinib-loaded exosomes (U87-Selu exo and A549-Selu exo), tumor volume and TGI were measured (Figure 11a,b). The tumor volume increased continuously up to 10 days in the saline group. In the A549-Selu exo-treated group, the tumor volume was increased, but the volume increase leveled off after 8 days. In the U87-Selu exo-treated group, the tumor volume was slightly increased, then decreased after 4 days. The biggest anticancer effect was produced by U87-Selu exo. However, it should be noted that the small number of replicates (*n* = 5) is a possible study limitation to confirm the measured tumor volumes. The TGI results were in good agreement with tumor volume measurements. TGI was 31.2% for saline, 55.9% for A549-Selu exo, and 99.8% for U87-Selu exo. Cleaved caspase-3, a tumor apoptosis marker, indicated that the U87-Selu exo-treated group exhibited the highest anticancer effect (Figure 11c). ki-67, a tumor cell growth marker, also showed that tumor growth was minimal in the U87-Selu exo-treated group (Figure 11c). Taken together, these results indicate that the U87-Selu exo-treated group experienced the strongest anticancer effects in a GBM xenograft model, suggesting that the high tumor-targeting effect of U87-derived exosomes plays an important role in cancer therapy.

### 3.7. In Vitro Anticancer Effect of U87-Selu Exo

#### 3.7.1. Apoptosis of U87-Selu Exo to U87MG Cells

**Figure 12:**
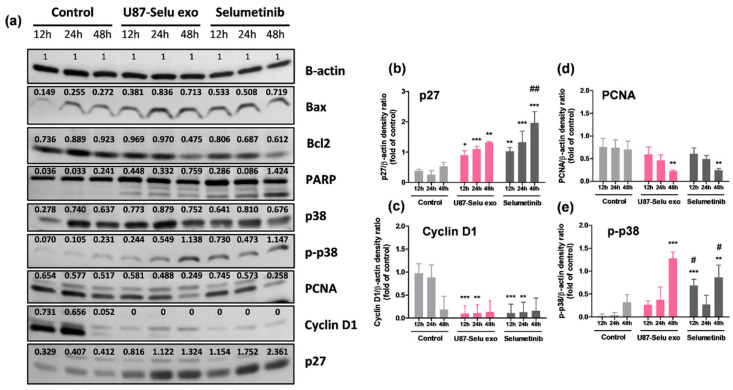
Apoptosis caused by U87-Selu exo in U87MG cells. (**a**) Western blot of U87MG cells treated with U87-Selu exo and selumetinib. (**b**) p27 expression levels at 12, 24, and 48 h. (**c**) PCNA expression levels at 12, 24, and 48 h. (**d**) Cyclin D1 expression levels at 12, 24, and 48 h. (**e**) p-p38 expression levels at 12, 24, and 48 h. * *p* < 0.05, ** *p* < 0.01, *** *p* < 0.001 vs. non-treated control for each time point; # *p* < 0.05, ## *p* < 0.01 vs. U87-Selu exo. *n* = 3.

Several studies have reported quantitative analyses to get better insights into the tumor microenvironment, including proteomics [41,42]. In this study, to evaluate its anticancer effect, we performed a Western blot analysis for apoptosis factors and observed the effect of U87-Selu exo on apoptosis (Supplemental Information, Figure S4). The levels of the cleaved form of Poly (ADP-ribose) polymerase (PARP) were increased by about 5.1-fold and 7.5-fold, respectively, compared with control, when U87MG cells were treated with U87-Selu exo and selu for 48 h (Figure 12a). The level of Bcl2 was decreased by about 0.8-fold compared with control when U87MG cells were treated with U87-Selu exo and selu for 48 h (Figure 12a). Because PARP and Bcl2 are useful markers of apoptosis [43,44], our results indicated that apoptosis occurred when the cells were treated with U87-Selu exo and selumetinib. By measuring the levels of p27, PCNA, cyclin D1, and p-p38, we confirmed that the proliferation of cancer was inhibited (Figure 12b–e). In the control group, the expression of cyclin D1 was downregulated at 48 h due to serum-free media starvation.

When cells were treated with U87-Selu exo and selumetinib for 24 h, the levels of p27 and p-p38 were increased by about 4.2, 5.1, 10.8, and 7.8 times in each group, respectively. In the case of PCNA and cyclin D1, the levels were decreased by about 0.6, 0.7, 0.1, and 0.1 times for each group (Table 1). Taken together, these results indicated that selumetinib and U87-Selu exo had anticancer effects on U87MG cells.

#### 3.7.2. Flow Cytometry

**Figure 13:**
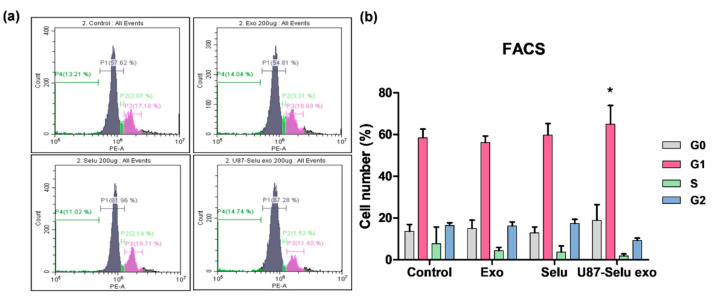
Cell cycle (G1 phase) arrest in U87-Selu exo as measured using flow cytometry. The harvested cells were subjected to PI staining and used for cell cycle analysis. (**a**) Cell cycle of each group; control, exo, Selu, and U87-Selu exo. (**b**) Number of cells in each phase. * *p* < 0.05 vs. non-treated control, *n* = 3.

To further evaluate the effects of U87-Selu exo on the apoptosis pathway by examining cell cycle arrest, fluorescence-activated cell sorting (FACS) analysis was performed. Cells were treated with 200 µg/µL of exo, Selu, or U87-Selu exo for 24 h, and then stained with PI. The control and exo-treated groups did not undergo cell cycle arrest (Figure 13a). Treatment with U87-Selu exo or Selu led to an increase in the G1 phase; the extent of arrest was about 11% in cells treated with U87-Selu exo. This increase was higher than what was observed in cells treated with Selu (Figure 13b). Because G1 phase arrest is associated with apoptosis of mesenchymal or epithelial cells [45,46], the FACS results showed that both U87-Selu exo and Selu induce G1 phase arrest, indicating apoptosis.

### 3.8. Anticancer Mechanism of U87-Selu Exo

**Figure 14:**
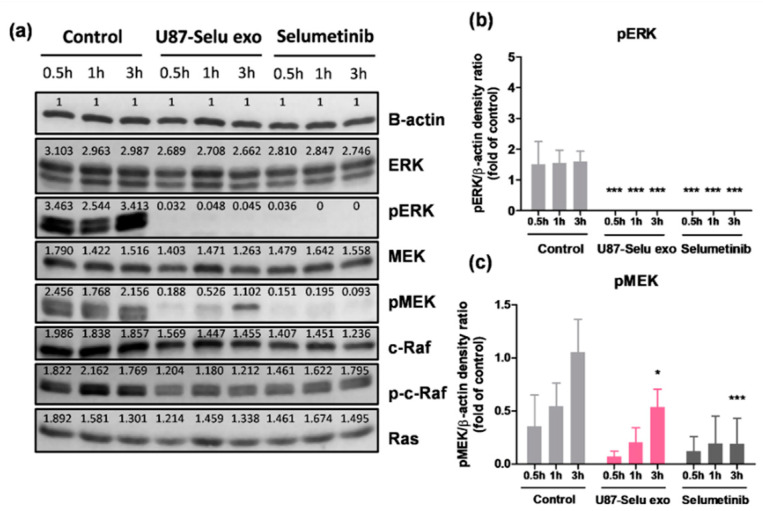
Anticancer mechanism of U87-Selu exo compared with selumetinib. (**a**) Western blot of each group treated for 0.5, 1, or 3 h. (**b**) pERK expression levels of control, U87-Selu exo, and Selu only. (**c**) pMEK expression levels of control, U87-Selu exo, and selumetinib only. * *p* < 0.05, *** *p* < 0.001 vs. non-treated control for each time point, *n* = 3.

Because U87MG cells, which were treated with U87-Selu exo, were stopped in the G1 phase and went through apoptosis, we further studied the mechanism of the U87-Selu exo (Supplementary Materials, Figure S5). Because selumetinib is known to be a pMEK inhibitor [47,48], we hypothesized that U87-Selu exo shows a similar anticancer mechanism as selumetinib. To confirm our hypothesis, we conducted Western blotting to evaluate the activation of intracellular signaling pathways. Compared with the control group, cells treated with U87-Selu exo or selumetanib showed downregulated expression of pMEK and pERK (Figure 14a–c). pMEK was lower by about 0.4- and 0.3-fold than the control group, and pERK was not detected at all in the U87-Selu exo-treated and selumetinib-treated groups. Through the expression of c-Raf, Raf, and Ras, we confirmed that U87-Selu exo and selumetinib are specific pMEK inhibitors. Taken together, these results confirmed our hypothesis that the anticancer mechanism of U87-Selu exo is like that of selumetinib, even after loading selumetinib into exosomes.

## 4. Conclusions

We demonstrated that selumetinib-loaded U87MG-derived exosomes (U87-Selu exo) can be used for targeted GBM (U87MG cell) therapy using their ability to target their parent cells. U87-Selu exo did not show any cytotoxicity to normal brain cells, even at high doses, and did not show any toxicity to the liver and kidney in vivo. Therefore, our findings indicated that U87-Selu exo has a specific antitumor effect on GBM with a U87MG origin. The non-toxicity of U87-Selu exo to normal brain cells and liver indicates that there are promising therapeutic options for the treatment of GBM. Furthermore, it is still challenging to demonstrate whether glioma-derived exosomes show an in vivo homing effect because the central nervous system has the blood-brain barrier. Therefore, future work is warranted to investigate the in vivo homing effect of glioma-derived exosomes using an appropriate brain tumor model, such as an orthotopic model. This future work is especially important in the clinical translation of glioma-derived exosomes for GBM treatment.

## Figures and Tables

**Table 1:** The expression of p27, PCNA, cyclin D1, and p-p38 when cells were treated with U87-Selu exo and Selumetinib for 24 h.

Expression of Factor	U87-Selu Exo (Fold)	Selumetinib (Fold)
p27	4.2 ± 0.3	5.1 ± 1.2
PCNA	0.6 ± 0.1	0.7 ± 0.1
Cyclin D1	0.1 ± 0.2	0.1 ± 0.2
p-p38	10.8 ± 6.5	7.8 ± 4.7

## Data Availability

The data presented in this study are available on request from the corresponding author.

## Supplementary Materials

The following supporting information can be downloaded at: https://www.mdpi.com/article/10.3390/pharmaceutics14051002/s1, Figure S1: Selumetinib standard curve. To calculate the loading efficiency of selumetinib into the exosomes, we observed selumetinib standard curve using UV-vis spectrometer; Figure S2: Fluorescent dye (DiI) labeling efficiency to U87 exo and A549 exo; Figure S3: Stability of U87-Selu exo in physiological pH (pH = 7.4) via exosome diameter; Figure S4: Western Blot full band of exosomal markers, non-exosomal maker for U87 exo, A549 exo, U87-Selu exo and A549-Selu exo and apoptosis, proliferation markers treated with non-treat control, U87-Selu exo (100 μg/μL) and Selumetinib (100 μg/μL) for 12 h, 24 h, 48 h; Figure S5: Western blot full band of mechasnism markers treated with non-treated control, U87-Selu exo (100 μg/μL) and Selumetinib (100 μg/μL) for 12 h, 24 h, 48 h.
