# Supplementary material for: Glioblastoma-Derived Exosomes as Nanopharmaceutics for Improved Glioma Treatment
Source: Pharmaceutics. 2022 May 6;14(5):1002. doi: 10.3390/pharmaceutics14051002 (PMC9145309; doi:10.3390/pharmaceutics14051002)
Supplement: Supplementary file 1 [file pharmaceutics-14-01002-s001.zip › pharmaceutics-1670950-supplementary.pdf]

Supplementary Materials: Glioblastoma-Targeted Exosome as Drug-Carrier for Enhanced Theranostic Agent by Exosome Targeting Effect

Hyeji Lee [1], Kanghye Bae [1], Ah-Rum Baek [2], Eun-Bin Kwon [3], Yeoun-Hee Kim [4], Sung-Wook Nam [5], Gang Ho Lee [6] and Yongmin Chang [1,5,*]

[1] Department of Medical Science, School of Medicine, Kyungpook National University, Daegu 41944, Korea; pba04052@naver.com (H.L.); hjbkh980708@naver.com (K.B.)
[2] Institute of Biomedical Engineering Research, Kyungpook National University, Daegu 41405, Korea; baxun@naver.com
[3] Korean Medicine (KM) Application Center, Korea Institute of Oriental Medicine, Daegu 41062, Korea; wrld2931@kiom.re.kr
[4] R&D Center, Etnova Therapeutics Corp., 124, Sagimakgol-ro, Jungwon-gu, Seongnam-si 13207, Korea; yhkim@etnova.co.kr
[5] Department of Molecular Medicine, School of Medicine, Kyungpook National University, Daegu 41944, Korea; nams@knu.ac.kr
[6] Department of Chemistry, College of Natural Sciences, Kyungpook National University, Daegu 41566, Korea; ghlee@knu.ac.kr
* Correspondence: ychang@knu.ac.kr

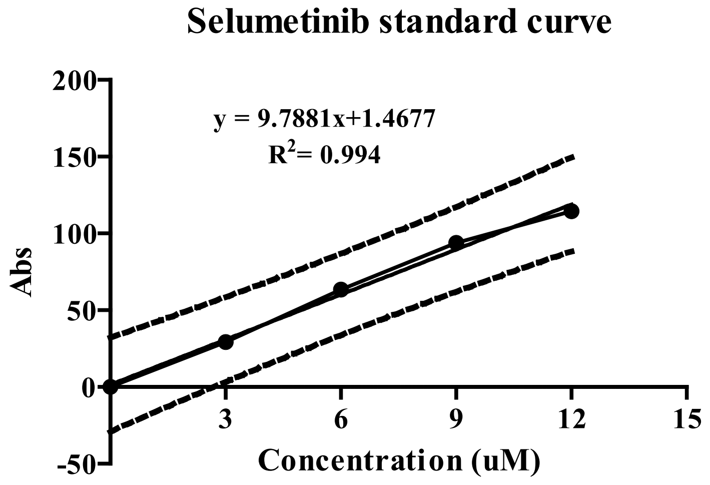

Figure S1. Selumetinib standard curve. To calculate the loading efficiency of selumetinib into the exosomes, we observed selumetinib standard curve using UV-vis spectrometer.

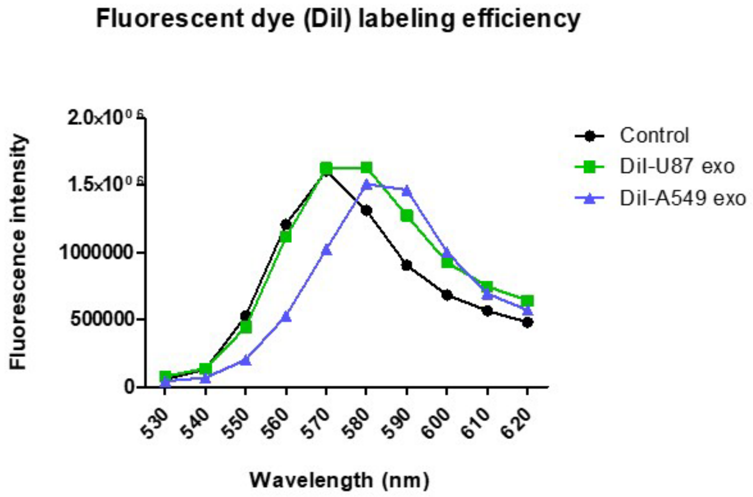

Figure S2. Fluorescent dye (DiI) labeling efficiency to U87 exo and A549 exo. The fluorescent dye was labeled to exosome to tracking the homing effect of exosomes and an excess of the dye was eliminated by exosome spin column (MW 3000). The fluorescent dye labeling efficiency was measured by UV-Spectrometer. Control was DiI in methanol following the protocol of manufacture.

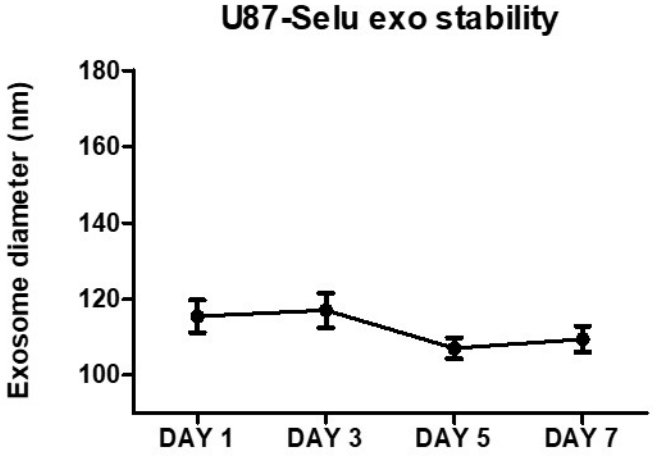

Figure S3. Stability of U87-Selu exo in physiological pH (pH = 7.4) via exosome diameter. U87-Selu exo were incubated with PBS (pH=7.4) and 37 ˚C for 1, 3, 5 and 7 days. Through the NTA results, the diameter of exosomes were stable during 7 days ranging from 108nm to 122nm.

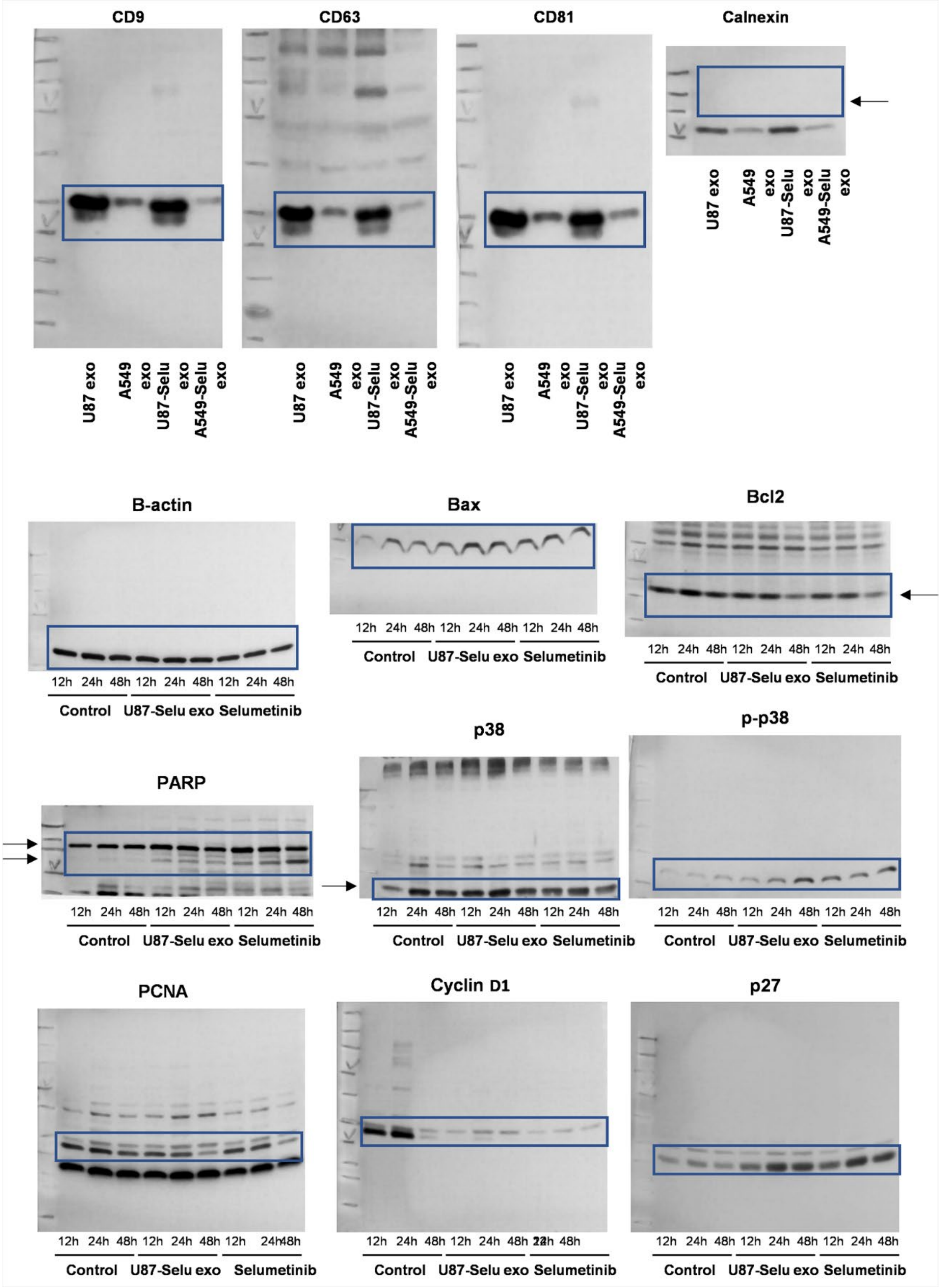

Figure S4. Western Blot full band of exosomal markers, non-exosomal maker for U87 exo, A549 exo, U87-Selu exo and A549-Selu exo and apoptosis, proliferation markers treated with non-treat control, U87-Selu exo (100μg/μl) and Selumetinib (100μg/μl) for 12hr, 24hr, 48hr.

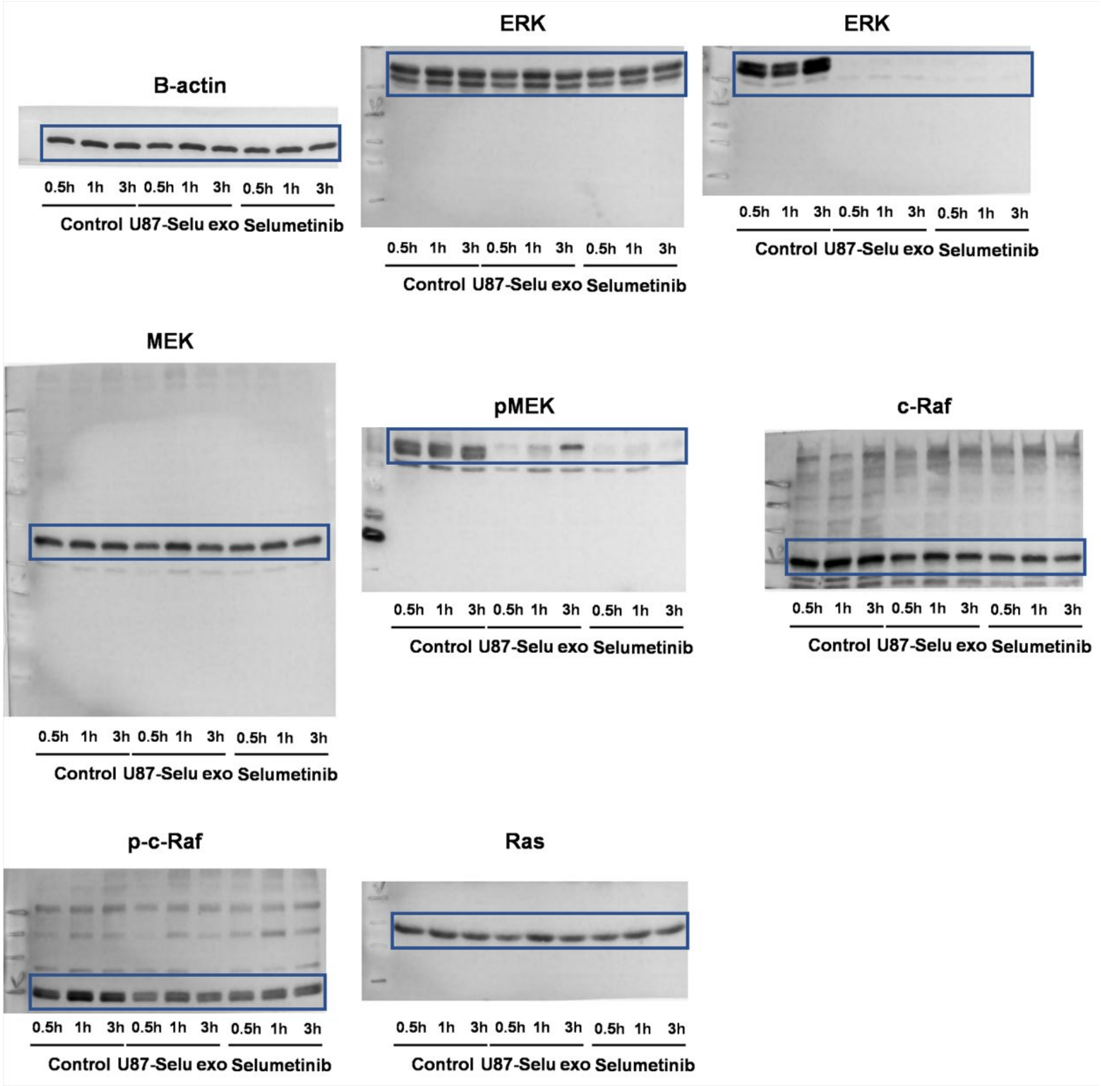

Figure S5. Western blot full band of mechasnism markers treated with non-treated control, U87-Selu exo (100μg/μl) and Selumetinib (100μg/μl) for 12hr, 24hr, 48hr.
